# Trajectory formation principles are the same after mild or moderate stroke

**DOI:** 10.1371/journal.pone.0173674

**Published:** 2017-03-22

**Authors:** Denis Mottet, Liesjet Elisabeth Henriette van Dokkum, Jérôme Froger, Abdelkader Gouaïch, Isabelle Laffont

**Affiliations:** 1 EuroMov, Univ. Montpellier, Montpellier, France; 2 Laboratoire Charles Coulomb, Univ. Montpellier, Montpellier, France; 3 Physical and Rehabilitation Medicine, University Hospital of Nimes, Nimes, France; 4 Laboratoire d’Informatique de Robotique et de Microelectronique de Montpellier, Univ. Montpellier, Montpellier, France; 5 Physical and Rehabilitation Medicine, University Hospital of Montpellier, Montpellier, France; University of California Merced, UNITED STATES

## Abstract

When we make rapid reaching movements, we have to trade speed for accuracy. To do so, the trajectory of our hand is the result of an optimal balance between feed-forward and feed-back control in the face of signal-dependant noise in the sensorimotor system. How far do these principles of trajectory formation still apply after a stroke, for persons with mild to moderate sensorimotor deficits who recovered some reaching ability? Here, we examine the accuracy of fast hand reaching movements with a focus on the information capacity of the sensorimotor system and its relation to trajectory formation in young adults, in persons who had a stroke and in age-matched control participants. We find that persons with stroke follow the same trajectory formation principles, albeit parameterized differently in the face of higher sensorimotor uncertainty. Higher directional errors after a stroke result in less feed-forward control, hence more feed-back loops responsible for segmented movements. As a consequence, movements are globally slower to reach the imposed accuracy, and the information throughput of the sensorimotor system is lower after a stroke. The fact that the most abstract principles of motor control remain after a stroke suggests that clinicians can capitalize on existing theories of motor control and learning to derive principled rehabilitation strategies.

## Introduction

Reaching with one’s hand is the building block of most of our movements with the upper-limb in everyday life. When we make rapid reaching movements, we have to trade speed for accuracy [1], and the way we do so is predicted by one of the most robust laws in motor control, the Fitts’s law [2]. The general idea behind this law is that a limited information throughput acts as a constraint on trajectory formation: the higher the accuracy requirement, the higher the amount of information to transmit and, consequently, the longer the time it will take to perform an accurate movement. In rapid reaching movements, feed-forward control is used to implement a fast initial impulse that, on average, rapidly moves the hand within the target [3]. Yet, due to signal-dependent noise in the motor command, a stronger initial impulse results in more endpoint variability [4,5], which might necessitate a corrective sub-movement. By optimally adjusting the average magnitude and duration of each noisy impulse used to generate a sub-movement, we can minimize the overall movement duration for the requested accuracy [6]. Nowadays, it seems undisputed that the way we trade speed for accuracy is an emergent feature of optimal trajectory formation in the face of noise in the sensorimotor system [7,8]. So, higher sensorimotor noise due to ageing is likely one possible cause of the strategic adaptations in sensorimotor control with age [9], whether resulting in lower information throughput [10–12] or not [13].

However, it is largely unclear if and how the same principles apply for movement generation with a damaged brain, such as it is the case after a stroke, for persons who recovered some reaching ability. Stroke results in higher sensorimotor noise compared to age-matched controls[14,15], and we have seen that the same is likely true for aging. Here, we want to disentangle the effects of ageing and of stroke. We address the organization of fast reaching movement after an unilateral stroke, in age-matched and in young control participants, with a focus on the information capacity of the sensorimotor system and its relation to trajectory formation, that is, in relation to the principles and laws that shape the geometrical and temporal features of our movements [16]. Stroke is the leading cause of long-term disability, despite many forms of neuroplasticity contributing to recovery [17]. Reaching movements post-stroke are characterized by coarse-grained force production with tremor [18]. It is also likely that moving outward in the homolateral space is more difficult, because gravity compensation devices are especially effective in this quadrant [19,20]. In addition, the unfolding of the hand trajectory in space and time is marked by more directional errors, stronger segmentation, and less overall speed [21–23]. However, for people with mild or moderate stroke, the lack of available force is not the main reason for the impaired control, which seems mainly related to unavoidable abnormal synergies [24,25]. All in all, the main characteristic of the motor output after a stroke seems to be a higher amount of noise, likely combined with a cost of the movement that varies as a function of its direction, especially for persons with severe sensorimotor deficits [26,27].

Therefore, we formulate three hypotheses. First, we hypothesize that increasing accuracy requirements will have a stronger impact on movement organization in people post-stroke and that this impact will be even stronger when moving outward in the homolateral space. Second, we hypothesize that both ageing and stroke will result in lower sensorimotor performance, yet with a stronger effect of stroke. Third, we hypothesize that people will follow the same principles of trajectory formation in the face of sensorimotor noise, whether they are young or aged and healthy, or suffering from stroke.

## Methods

### Participants

A group of 19 adults with stroke (aged 61.0 ±13.8, 9 male) performed the task with their paretic hand. Inclusion criteria were (1) unilateral stroke at least one month before, (2) capacity to hold a pen, with more than 2 at the Enjalbert test [28], and (3) capacity to actively extend the elbow up to 135° in the horizontal plane. Exclusion criteria were (1) neglect (Catherine Bergego scale > 0/30 [29]) and (2) cognitive deficits (mini mental state examination < 25/30 [30]). Table 1 provides more detailed information about the patient group.

**Table 1:** Characteristics of the hemi-paretic group.

	Gender	Age	MAS	Month since stroke	Brain lesion side	Brain lesion type	Brain lesion location	Enjalbert score
P01	F	48.1	0	2	R	I	Middle cerebral artery	5
P03	F	57.8	1+	3	L	H	Thalamic capsule	3
P04	F	53.6	1	35	L	I	Superficial middle cerebral artery	2
P05	M	70.4	0	4	R	I	Superficial middle cerebral artery	4
P06	F	72.6	0	5	L	H	Putamen	2
P07	M	61.9	1	3	L	I	Internal Capsule	4
P09	F	52.2	0	5	R	I	Internal Capsule	2
P10	M	58.5	1	2	L	I	Middle cerebral artery	3
P11	M	57.9	0	4	R	I	Middle cerebral artery	2
P12	M	69.4	1	2	L	H	Capsulo-thalamic	3
P13	F	67.3	0	2	L	I	Middle cerebral artery	4
P14	M	69.1	0	10	R	H	Capsulo-thalamic	5
P16	M	64.9	0	2	L	I	Para middle cerebral artery	6
P20	F	23.0	1	5	L	H	Frontal parenchymatic	2
P21	M	74.0	1	63	R	I	Temporal lobe	2
P22	M	71.6	0	3	R	H	Thalamic capsule	3
P23	F	75.1	0	4	R	I	Middle cerebral artery	3
P25	F	37.3	0	5	L	I	Middle cerebral artery	2
P29	F	74.5	0	4	R	I	Middle cerebral artery	5

Brain lesion side is right of left (R/L). Brain lesion type is ischemic or hemorrhagic (I/H). MAS stands for Modified Ashworth Scale for the assessment of spasticity in elbow flexors from 0 (normal) to 4 (rigid). The Enjalbert test classifies prehension from 0 (no motricity) to 6 (sub-normal).

A group of 13 age-matched healthy participants (aged 65.2 ±9.5, 6 male) and a group of 19 healthy young adults (aged 25.7 ±2.5, 14 male), with no history of neurological disease, performed the task with their dominant hand. The young group provided the reference of the best possible performance at the task, which allowed to disentangle the effects of ageing and of stroke, so to complement previous works [14,15].

All participants had normal or corrected-to-normal vision.

All study procedures were approved by the Ethics Committee of Nimes, France (n° 2010- A0059633) and executed at the Physical and Rehabilitation Medicine Departments of the University Hospital of Nimes and of Montpellier. All participants gave written informed consent before inclusion. All procedures complied with the ethical standards outlined by the Declaration of Helsinki.

### Procedure

Participants sat in front of a graphic tablet (Wacom Intuos4 XL, A3), facing a screen (Apple, 17 inch) located about 60 cm from their eyes, and with their belly in contact with the edge of the graphics tablet. A displacement of 20 cm of their hand on the graphics tablet corresponded to a 16 cm displacement of the cursor on the screen (approximately 16° of visual angle). Participants could see their own hand movement on the graphics tablet with peripheral vision. We did not use devices for trunk restriction or upper-limb support. For some patients, the pen was strapped to the fingers of the paretic hand to facilitate the grip.

**Fig 1:**
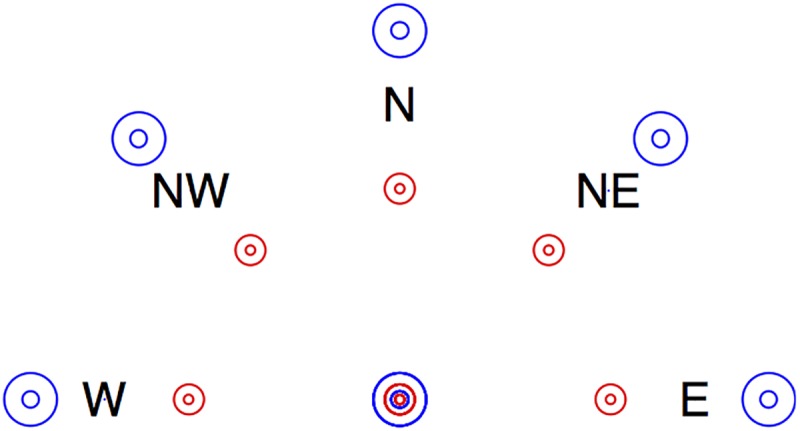
Disposition of the targets. The task was to successively reach each target in a fixed order. The letters W, NW, N, NE, E refer to movement orientation coded according to the rose of the winds. Only one target was present on the screen at a time. No click was required: the reaching was successful when staying within the target for 1 s (see Fig 2). After reaching the central target (large blue circle), the trial started with the outward reaching to the target at the largest distance and lowest difficulty (i.e., large blue target to the west). Each outward reach was followed with an inward reach to the central target. The whole sequence of reaching was outward then inward, along directions from west to east, at distances of 175 and 100 mm (blue and red circles) and at large and small targets, whose size was equal to 14.28% and 4.62% of the distance.

After familiarization, participants had to perform two trials separated by a rest period. Each trial consisted of a series of 40 reaching movements at targets appearing one after the other, in a fixed order described in Fig 1. Participants were instructed to perform the whole sequence as fast as possible.

In total, each participant performed 80 reaching movements, which corresponded to 20 task conditions (5 orientations × 2 directions × 2 indexes of difficulty) repeated 2 times in each of the 2 trials. Motion orientation was coded according to the rose of the winds: west, northwest, north, northeast and east (W, NW, N, NE, E see Fig 1). The same index of difficulty (3.80 and 5.43 bit) was obtained for reaching distances of 175 and 100 mm by setting target size proportional to distance (14.28% and 4.62%). Preliminary tests revealed that random order, higher average difficulty or more reaching movements was not possible in a single experimental session for persons with stroke.

### Data analysis

**Fig 2:**
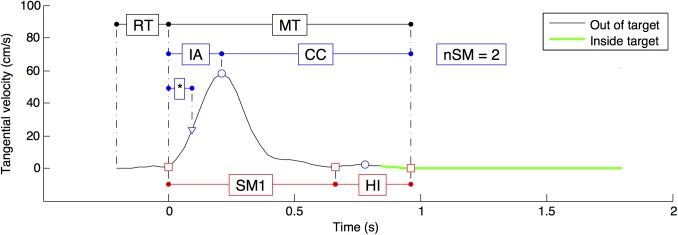
Typical tangential velocity profile showing the method for parsing a reaching movement. The reaching was successful when the cursor remained within the target for 1 sec (no click required). Red circles indicate velocity minima. Blue squares indicate velocity maxima. The blue triangle represents peak acceleration. The figure illustrates the durations of the reaction time (RT), movement time (MT), time to peak acceleration (*), first sub-movement (SM1), homing-in (HI), initial adjustment (IA) and current control phase (CC). The beginning of the MT is the last velocity minimum inside the previous target. The end of the first sub-movement is the first velocity minimum. The end of the MT is the first velocity minimum that is inside the target. The number of sub-movements (nSM) is the number of velocity maxima.

Movements performed with the left hand were flipped so that a reaching to the east always corresponded to a reaching to the side of the active upper-limb. Position time series were recorded every 30 ms and low pass filtered at 15 Hz. Each reach movement was parsed using hand velocity profile (see Fig 2). The appearance of the target defined the Go signal. The movement time (MT) was the duration between when the pointer left the start target and the first velocity minimum that was within the target. The first velocity peak was used to separate an *initial adjustment* (IA) phase and a *current control* (CC) phase [1,31,32]. The first velocity minimum was used to estimate the end of the first sub-movement (end of SM1 in Fig 2), which includes IA and the first part of the deceleration, up to the first re-acceleration. At these marked points within a movement time (Fig 2), we analyzed the percentage of movement distance and time that was covered to get a numerical estimate of the shape of the trajectory in time and space [3,33,34]. The number of sub-movements (nSM) was assessed using the number of velocity maxima [23].

### Information capacity

When reaching at a target, the relative accuracy (namely, the ratio of target distance D over target width W) determines the index of difficulty of the task, ID = log_2_(2D/W). The ID measures the amount of information that the sensorimotor system must transmit during execution of the task, by direct analogy to Shannon’s theory about communication of information over a noisy channel [35].

In fast and accurate reaching, more accuracy implies more information and, consequently, the movement time becomes longer to accommodate the higher amount of information to transmit. This rationale gave rise to a famous quantitative relation in human motor control, the Fitts’s law, which predicts that the movement time MT will be a linear function of the index of difficulty ID [2,3,6]. In the equation MT = *a* + *b* ID, the slope (*b*) captures the informational aspects of the performance, and the intercept (*a*) aggregates the non-informational aspects in the MT vs. ID relation [36]. The inverse of the slope (1/*b*) quantifies the information throughput [2,36,37]. The intercept (*a*) quantifies the minimum time for a movement without accuracy, which makes sense, due to the limited acceleration capabilities of any effector [38]. Note that the values of the parameters (*a*) and (*b*) are specific to each individual person, but they also depend on the device/effector used to perform the task [39].

### Statistical analysis

The effects of the experimental manipulation were assessed for each dependent measure using an ANOVA with one between-group factor (G: Young/Age-matched/Patient) and three within-group factors: orientation (ORI: W/NW/N/NE/E), direction (DIR: Outward/Inward) and index of difficulty (ID: 3.80/5.43). Effect size was estimated with generalized eta squared ($\eta_{G}^{2}$), which is well adapted to mixed within-between design [40]. Statistical significance was set to alpha < .01, with Greenhouse & Geisser corrected probabilities for the ORI factor. Effects with $\eta_{G}^{2}$ < 0.01 were deemed too small to be considered [40]. Post hoc multiple mean comparisons with Tukey contrasts were used to complement the ANOVA.

For the movement time, the data were not normally distributed. As a consequence, it is recommended to use non-parametric tests to assess the effects of the experimental factors. However, the ANOVA is robust to normality violation provided that the distributions are similar in shape [41], and we moreover found that the ANOVA and the non-parametric tests yielded similar conclusions. Consequently, for the sake of simplicity, we report the ANOVA results for all variables.

## Results

### Movement time and the Fitts’s law

The movement time was longer for participants with stroke (*F*(2, 48) = 35.32, *p* = 0.0000, $\eta_{G}^{2}$ = 0.67), increased with the index of difficulty (*F*(1, 48) = 5.42, *p* = 0.0000, $\eta_{G}^{2}$ = 0.03), and this increase was stronger for patients (*F*(2, 48) = 10.02, *p* = 0.0003, $\eta_{G}^{2}$ = 0.03). Post-hoc comparison indicated that the mean movement time for participants with stroke what higher than for the young and age-matched control groups, which did not differ significantly (respectively, *p* = 0.0000, *p* = 0.0000 and *p* = 0.7880).

**Fig 3:**
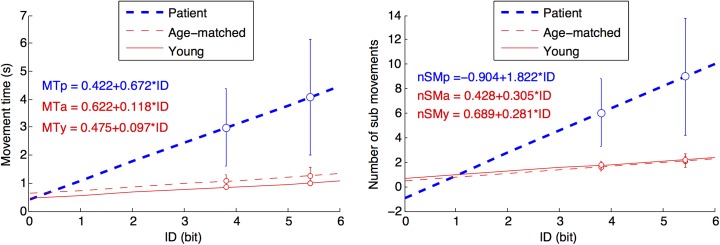
Movement time (MT) and number of sub-movements (nSM) as a function of index of difficulty (ID). Each point in the figure corresponds to 40 reaching movements multiplied by the number of participants in the group, for the patients with stroke (blue line) and the aged-matched and young adults (thin red lines). Errors bars indicate standard deviations between participants. The difference in slope between the groups indicates that the effect of the informational load (ID) is about 5–7 times stronger for persons with stroke, for the MT and for the nSM (see also Table 2). There is no significant difference in intercept. The number of sub-movements is likely the main determinant of the lower information throughput in persons with stroke.

As a consequence, the slope of the Fitts’s Law was higher after a stroke (Fig 3, left panel), which indicates that the information throughput was lower (Table 2). The consequences of stroke resulted in a significant slow down of 6.42 bits/sec (82% less than age matched). The intercept of the Fitts’s Law did not vary significantly among groups (*F*(2, 48) = 0.17, *p* = 0.8391, $\eta_{G}^{2}$ = 0.00).

**Table 2:** Average values of the main variables for the three experimental groups.

Variable	Age-matched	Young	Patient
Movement time (sec)	1.17	0.92	3.53
Fitts’s slope (sec/bit)	0.118	0.097	0.672
Throughput (bit/sec)	8.47	10.30	1.49
Sub Movements (number)	1.84	1.99	7.52
Time in IA (%)	28.67	27.87	17.48
Distance in IA (%)	39.27	43.20	22.49
Error in distance at the end of SM1 (%)	7.55	8.26	56.25
Error in direction at the end of SM1 (°)	1.38	1.62	8.72

For all variables in the table, multiple comparisons with Tukey contrasts indicate that the means of the Age-matched and Young groups do not differ, but differ from the means of the Patient group.

We did not find significant effects involving the DIR and ORI factors.

### Feed-forward and feed-back control

To better understand the control processes at work to accommodate the speed and accuracy constraints, we divided each reaching movement into two phases corresponding to a dominant role of different control processes [1,3]: feed-forward in the *initial adjustment phase* and feed-back in the *current control phase* (see Fig 2).

#### Movement distance and time during the initial adjustment phase

The percentage of movement time in the initial adjustment phase was lower for participants who had a stroke (*F*(2, 48) = 27.17, *p* = 0.0000, $\eta_{G}^{2}$ = 0.38), when moving outward (*F*(1, 48) = 27.75, *p* = 0.0000, $\eta_{G}^{2}$ = 0.03), when the ID was high (*F*(1, 48) = 151.74, *p* = 0.0000, $\eta_{G}^{2}$ = 0.10), and when moving closer to the North orientation (*F*(4, 192) = 8.48, *p* = 0.0000, $\eta_{G}^{2}$ = 0.02).

The percentage of the distance to the center of the target that was covered in the initial adjustment phase was lower for participants who had a stroke (*F*(2, 48) = 77.85, *p* = 0.0000, $\eta_{G}^{2}$ = 0.62) and when moving outward (*F*(1, 48) = 110.09, *p* = 0.0000, $\eta_{G}^{2}$ = 0.08), the latter effect being stronger when moving along the West and East orientations (IO:ORI: *F*(4, 192) = 9.83, *p* = 0.0000, $\eta_{G}^{2}$ = 0.03).

**Fig 4:**
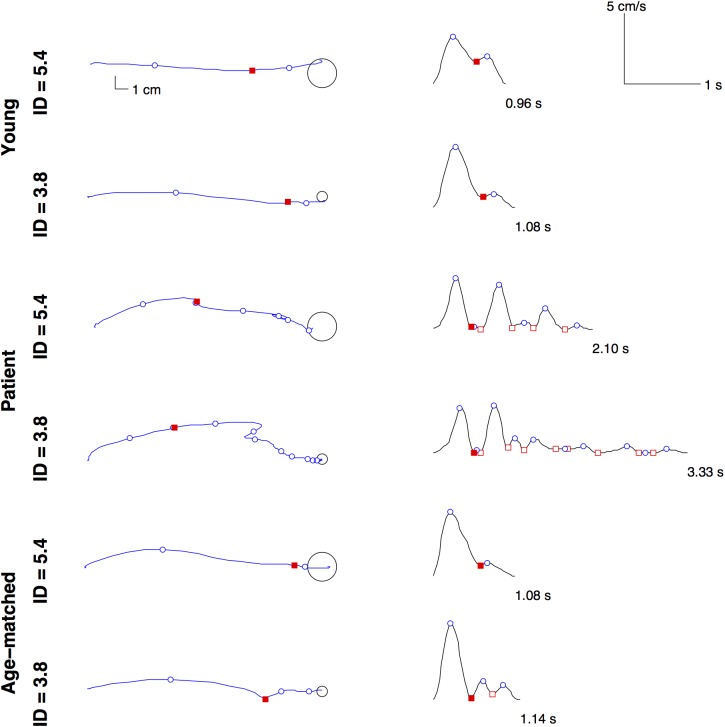
Typical movement patterns. The figure shows typical trajectory and velocity profile during an outward reaching toward the East for persons with stroke, age-matched and young control participants, as a function of the index of difficulty (ID). The trajectory ends at the rightmost circle that represents the target. The movement time is reported at the end of each velocity profile. Blue circles represent velocity peaks used to count the sub-movements. Red squares indicate velocity minima, and the filled square emphasizes the end of the first sub-movements (SM1 in Fig 2). The initial adjustment phase is from the beginning of the movement up to the first blue circle (IA in Fig 2). The initial adjustment phase, driven by feed-forward control, is shorter after a stroke, both in space (trajectory) and in time (velocity profile). All in all, persons with stroke use shorter and more numerous sub-movements, and they need more time to complete a movement with similar accuracy.

The initial adjustment phase, driven by feed-forward control, was proportionally shorter—in space and in time—after a stroke, as illustrated in Fig 4, where typical examples of trajectories and velocity profiles are provided.

#### Errors in direction and distance at the end the first sub-movement

The previous results converge to indicate that persons with stroke relied far less on feed-forward control when reaching with speed and accuracy. To better understand why, we investigated the efficiency of the first sub-movement (SM1 in Fig 2). We analyzed the error at the end of the first sub-movement, which should be zero for an ideal movement without corrections. We computed the error in direction and in distance. The error in distance was the Euclidian distance from the current position to the center of the target. The error in direction was the angle between three points: the current position, the center of the target, and the position at the beginning of the movement.

At the end of the first sub-movement, the mean error in distance to the center of the target was about doubled for persons who had a stroke (*F*(2, 48) = 88.68, *p* = 0.0000, $\eta_{G}^{2}$ = 0.66, see Table 2), was increased when reaching outward (*F*(1, 48) = 88.08, *p* = 0.0000, $\eta_{G}^{2}$ = 0.07) and the latter effect was less strong when moving along the North and North-West directions (*F*(4, 192) = 5.60, *p* = 0.0003, $\eta_{G}^{2}$ = 0.02). The error in distance at the end of the first sub-movement tended to be less for higher ID, but this effect was very weak (*F*(1, 48) = 5.00, *p* = 0.0301, $\eta_{G}^{2}$ = 0.00).

At the end of the first sub-movement, the mean angular error in direction was about 5 times larger in participants with stroke (*F*(2, 48) = 45.59, *p* = 0.0000, $\eta_{G}^{2}$ = 0.32, see Table 2) and increased when reaching outward (*F*(1, 48) = 12.31, *p* = 0.0010, $\eta_{G}^{2}$ = 0.01) especially for patients (*F*(2, 48) = 11.00, *p* = 0.0001, $\eta_{G}^{2}$ = 0.02). The angular error at the end of the first sub-movement did not depend on the ID (*F*(1, 48) = 0.40, *p* = 0.5297, $\eta_{G}^{2}$ = 0.00).

#### Trial-to-trial within-participant variability during a movement

The previous results indicate that, after a stroke, the first sub-movement results in larger errors, both in direction and in distance. To better understand how participants coped with the final accuracy requirements in spite of noise in their movement production, we analyzed how variable was their movements at peak acceleration, peak velocity, peak deceleration, and at the end of the movement [3]. The dependent variable was the within-participant standard deviation of the displacement in the primary direction of the movement at repetitions of the same experimental condition [3].

At peak acceleration, within-participant variability was higher for the persons with stroke (F(2, 48) = 30.41, p = 0.0000, $\eta_{G}^{2}$ = 0.32).

At peak velocity, within-participant variability was higher for persons with stroke (F(2, 48) = 31.57, p = 0.0000, $\eta_{G}^{2}$ = 0.13).

At peak deceleration, within-participant variability was higher for persons with stroke (F(2, 48) = 56.37, p = 0.0000, $\eta_{G}^{2}$ = 0.30) and when reaching outward (F(1, 48) = 25.58, p = 0.0000, $\eta_{G}^{2}$ = 0.02).

At the end of the reaching movement, within-participant variability was higher for persons with stroke (F(2, 48) = 13.71, p = 0.0000, $\eta_{G}^{2}$ = 0.09). Because the tolerance was higher for low ID, within-participant variability was higher for lower ID (F(1, 48) = 108.90, p = 0.0000, $\eta_{G}^{2}$ = 0.13), and the G×ID interaction indicated that patients exploited better the larger tolerance (G:ID F(2, 48) = 14.44, p = 0.0000, $\eta_{G}^{2}$ = 0.04).

**Fig 5:**
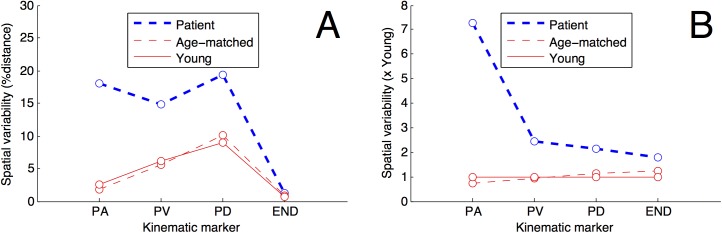
Movement variability within a reaching movement. The figure presents trial-to-trial within-participant standard deviation of the displacement in the primary direction of the movement at peak acceleration (PA), peak velocity (PV), peak deceleration (PD), and end of the movement (END) for the patients with stroke (blue line) and the aged-matched and young adults (thin red lines). Each point in the figure corresponds to 40 reaching movements multiplied by the number of participants in the group. In panel B, data are in percentage of the Young group, used as the reference. Spatial variability is higher for patients at all kinematic markers, but do not differ significantly between aged and young healthy adults.

All in all, within-participant variability was systematically higher for people with stroke than in both control groups, and the difference was especially strong at peak acceleration (Fig 5), which suggests that feed-forward control was not enough to cancel out higher noise levels in early movement production for people with stroke.

#### Accuracy as a consequence of the number of feed-back loops

The previous results indicate that, after a stroke, variability is larger in the initial adjustment phase, especially early in the movement, when only feed-forward control can account for trajectory formation. Consequently, greater variability in the initial adjustment must be compensated by larger and/or more numerous corrections in the current control phase [1,3,31,42]. Following this reasoning, we investigated the number of velocity peaks, to get a rough but robust estimate of the number of sub-movements, which is desirable when dealing with patient data [23].

The number of sub-movements was higher for participants with stroke (*F*(2, 48) = 37.24, *p* = 0.0000, $\eta_{G}^{2}$ = 0.60), and increased with the index of difficulty (*F*(1, 48) = 26.10, *p* = 0.0000, $\eta_{G}^{2}$ = 0.04), but it increased stronger in patients (*F*(2, 48) = 11.46, *p* = 0.0000, $\eta_{G}^{2}$ = 0.04) as illustrated in Fig 3, right panel.

**Fig 6:**
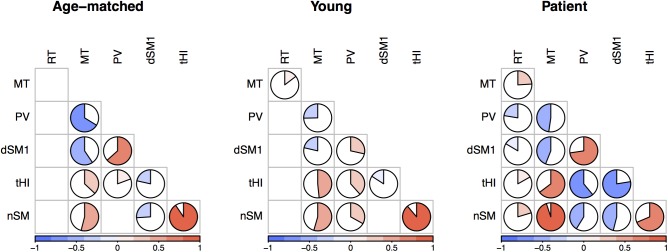
Correlation matrices between reaction time (RT), movement time (MT), peak velocity (PV), relative distance of the primary sub-movement end (dSM1), relative duration of homing-in (tHI) and number of number of sub-movements (nSM), for the three experimental groups. Only significant correlations are illustrated. The colored pie figures the value of the Pearson’s r, using the color code from the bar at the bottom. In all groups, higher PV result in longer dSM1 and higher nSM result in longer tHI and longer MT. Because the strongest correlation is between the nSM and the MT, and especially in the patients group, it is likely that the nSM is the primary cause of MT changes after a stroke.

We also investigated the relations between the main movement’s characteristics [33,34], that is, between reaction time (RT), movement time (MT), peak velocity (PV), relative distance during the first sub-movement (dSM1), relative duration of homing-in (tHI) and number of number of sub-movements (nSM). Correlations between these variables (Pearson’s r values) are summarized in Fig 6. As expected, in all groups, higher peak velocity resulted in larger distance during the primary sub-movements (i.e., positive correlations between PV and dSM1), though less strongly in young participants. Similarly, in all groups, primary sub-movements that were shorter in space allowed more time for homing-in (i.e., negative correlation between dSM1and tHI), and this correlation was especially strong for persons with stroke. As expected again, the homing-in phase lengthened with the number of corrections (i.e., positive correlations between tHI and nSM, though less strongly after a stroke), with the consequence that the movement time also lengthened when the percentage of homing-in was high, due to a larger number of corrective sub-movements (i.e., positive correlations between MT, tHI and nSM). Because the strongest correlation is between the nSM and the MT in persons with stroke, it is likely that the nSM is the primary cause of changes in MT for persons with stroke.

## Discussion

We addressed the accuracy of fast reaching movements after mild or moderate stroke, with a focus on the information capacity of the sensorimotor system and its relation to trajectory formation. By comparing a group of participants with stroke, age-matched and young control participants in a single experiment, we could disentangle the effects of stroke and of ageing.

In agreement with our first hypothesis, we found that increasing accuracy requirements had a stronger impact on movement organization in people with stroke, with a lower information throughput of the sensorimotor system, compared to age-matched and young. People with stroke made larger errors in direction and distance at the end of the first sub-movement, and compensated for this higher variability in movement production with a shorter first sub-movement indicating a strong minimization of feed-forward control, and with more numerous feed-back loops at the cost of longer movement times. Conversely to our initial hypotheses, we found that ageing did not result in significantly lower sensorimotor performance in this particular task, and we also found that the impact of stroke was not significantly stronger in the homolateral space. Finally, in agreement with our last hypothesis, we found that people in all groups follow the same principles of trajectory formation to move with speed and accuracy.

### Informational and non-informational aspects of motor performance after a stroke

After a stroke, movements with the paretic limb are jerkier [23], and globally slower for the same task difficulty [14]. Here, we found that the information throughput was 5–7 times lower after a stroke (6.42 bits/sec, 82% less than age-matched), but did not differ between the young and aged control groups. We also found that the minimum time for a movement without accuracy was similar.

We expected the lower information throughput after a stroke (illustrated by the higher slope for the patients’ group in Fig 3, right panel) as it was already reported for persons with chronic stroke compared to age-matched people [14]. We also expected a lower information throughput due to ageing [10,11]. Conversely, we found that the small decrease in information throughput with age did not reach significance. This was reported when varying ID by varying target size only [13], and the changes in ID in our experiment were mainly due to changes in target size. Consequently, we conclude that stroke induces a strong decrease in information throughput, but that the decrease due to ageing is far weaker, hence was too small to be detected in our experiment.

We did not expect a similar minimum time for a fast movement without accuracy in the three groups (illustrated by the similar intercept in Fig 3, right panel). Conversely, we reasoned that the limited force after a stroke would cause an increase in the minimum time for a fast movement without accuracy [38], hence should result in a higher intercept for patients in Fig 3. A higher intercept was reported for the paretic arm after a stroke in comparison to age-matched controls, and also within participant, in comparison to the less affected arm [14]. In comparison to the present experiment, we note that the difference in intercept was obtained with doubled movement distances, and was mainly true for the most severely impaired participants [14]. Consequently, we interpret the present lack of significant difference with caution, because extrapolations are very sensitive to errors (ID = 0 is outside the ID range) and because the between-participants standard deviations were high for persons with stroke.

All in all, persons after a stroke are clearly more sensitive to accuracy constraints than age-matched and young adults, but we cannot conclude that mild or moderate stroke influences the minimum time for a fast movement without accuracy. This calls for analyses accounting for the organization inside a movement time, to get a better perspective on trajectory formation, especially when comparing participants after a stroke and age-matched control participants.

### Trajectory formation after a stroke: feed-forward vs. feed-back control

Since the seminal work of Woodworth, better understanding the relative part played by feed-forward and feed-back control in fast and accurate movement became important [1,31]. With the boundary between the two phases set at peak velocity, the asymmetry of the velocity profile provides a good estimate of the feed-forward vs. feed-back ratio [43]: More asymmetry indicates a deceleration that is longer and devoted to the corrections that ensure to meet the accuracy constraints, which is classically reported for discrete [39] as well as for rhythmical movements [44]. Here, we found that the percentage of time under feed-forward control was higher when the ID was low, in the three groups.

The novel result is that, on average, the percentage of distance covered under feed-forward control was halved after a stroke and lasted for a smaller part of the movement time (Table 2) while within-participant variability was 7 times higher at peak acceleration and (Fig 5) compared to both young and age-matched control participants. These results indicate that, after a stroke, feed-forward control was not enough to cancel out higher noise levels in early movement production so that persons with stroke relied less on feed-forward to achieve the requested spatial accuracy. Moreover, we found that participants with stroke made 4 times larger errors than healthy at the end of the first sub-movement, which replicates the finding that directional errors increase with impairment after a stroke [32,45]. A computational model of cortical damage and its consequences on arm reaching movements revealed how the errors could be correlated with the loss of neurons [46,47]. In addition, participants with stroke are quickly confronted with fatigue [48], and fatigue contributes to increasing signal-dependent noise [49]. All this translates into higher noise levels, resulting in a larger disparity between the planned and the actual movement. As a consequence, because persons with stroke experience a more variable link between what they aim and what they do, persons with stroke have no option but minimizing feed-forward control to accommodate the accuracy constraints.

Moreover, as the decreased signal-to-noise ratio increases the probability of errors, persons with stroke have to wait for feed-back information to run (a series of) corrections to reach the target. As illustrated in Fig 4, it looks like persons with stroke reach the target with a concatenation of non-overlapping sub-movements, that is, with sub-movements separated by pauses. We checked for the occurrence of pauses by counting the number of sub-movements separated by a velocity lower than 1% of peak velocity. The number of pauses within a reach movement was more than doubled in persons with stroke (i.e., 1.35 vs. 0.54 and 0.55, F(2, 48) = 5.43, p = 0.0075, $\eta_{G}^{2}$ = 0.07). Hence, more than young and age-matched adults, participants with stroke have to wait for the end of a sub-movement before starting the next.

Finally, we point out that most of the changes in movement production that we observed seem related to a lower signal to noise ratio. We found that when the ID is high, all people used a shorter first sub-movement and an increased number of sub-movements to ensure that the required accuracy is met, which are typical features of the “play-it-safe” strategy [3]. The “play-it-safe” strategy was evidenced in older adults, but also in young adults when visual feedback is compromised [33,34]. We also replicated the finding that, in the face of an increase in task demands, participants decreased feed-forward control and favored a feed-back based strategy irrespective of age [33], and we extend this result to participants with mild to moderate stroke. However, our results also indicate that within-participant variability at peak acceleration is higher for persons with stroke when it is similar for young and aged participants (Fig 5). This result is important, because it reveals likely the main reason why persons with stroke are always using less feed-forward control: this is because they are not able to produce reliable muscular forces. Also, stroke results in more directional errors, stronger segmentation, less overall speed [21–23], it impairs the ability to implement internal models used for anticipatory control of arm movement [50], and all this might be due to the high noise level.

We conclude by pointing out that all these findings are coherent with the demonstration that signal dependent noise in movement production determines movement organization, via open-loop optimization [4] and optimal of feed-back control [8,51]. This is an important point, which indicates that, even though the movement appears jerky and disorganized after a stroke, trajectory formation relies, at least in part, on the same organizing principles as in healthy persons.

### Information capacity does not depend on motion orientation

In healthy adults, movement time is lower when the orientation of the movement is perpendicular to the forearm [52,53] and biomechanical manipulations of the force/mass ratio can influence the shaping of reaching trajectories [54]. Here, the lack of significant effect of the orientation factor in the control groups is, we think, due to two combined causes. First, the intensity of the orientation effect is very small, with $\eta_{P}^{2}$ about 0.01, here as in [52]. Second, reaching in healthy adults is largely planned independently of position and orientation of the end-effector in space [53,55]. Thus, we conclude that the effect of the orientation was here too small to be detectable.

When reaching after a stroke, movements to the north-east quadrant are often more difficult to control, and it is in this quadrant that gravity compensation devices are the most effective [19]. Here, we did not find systematic effects of the orientation of the reaching movements, which is counterintuitive from a clinical perspective. It is likely that this lack of variations of movement unfolding according to the direction of movement is because the range of motion was short and without restrictions of trunk compensations, and because we did not include patients with severe deficits, who are more sensitive to the position of the target in the workspace [26]. Thus, we conclude that the control of the reaching after a stroke is largely independent of the orientation and direction of the movement for persons without severe sensorimotor deficits.

Our findings indicate that, in all groups, the influence of biomechanical constraints was an order of magnitude lower than the influence of informational constraints. We cannot exclude that this hierarchy of constraints is specific to the present reaching task in 2D, because the orientation effect is absent in all the groups. However, the fact that the initial adjustment phase is shorter when reaching outward indicates an effect of the biomechanical constraints on trajectory formation [52,54], and this effect was similar in all three groups.

From a theoretical perspective, the fact that the information capacity of the sensorimotor system does not depend on the biomechanical constraints is predicted by the abstract nature of the informational approach [2,36,38,56]. Moreover, trajectory formation is largely independent of the orientation in space in which the trajectory is implemented in healthy adults [55,57] and after a stroke as well [45]. It is thus logical that movement orientation had a minimal influence on trajectory formation, especially when compared to the influence of accuracy. Task constraints lie in the forefront and biomechanical constraints lie in the background [52].

The fact that the same hierarchy of constraints applies for sensorimotor control in healthy adults and after a stroke is a novel important idea brought about by our work. The presence of the same hierarchy of constraints in the face of increased noise after a stroke suggests that these abstract principles are preserved after a stroke, at least for mild and moderate deficits. Although this remains to be confirmed with dedicated experimental work, the promise that the most abstract principles of sensorimotor control and learning remain after a stroke is good news. Therapists can capitalize on such abstract principles for the design of effective rehabilitation programs that drive neural plasticity towards better and faster recovery [17].

### Limitations of this study

We used only two ID values to assess the slope and intercept in the relation ID-MT and ID-nSM that are presented in Fig 3. This hinders the possibility to check that the linear model is correct, which should be the case for the movement time MT in young [6] and age-matched control participants [13], as well as in persons with stroke [14,15,32], but is only suspected for the number of sub-movements nSM [14].

We used velocity minima as the only criterion parse a movement time into sub-movements. Even though this simple method is widely used [9,23,33,34,58], it does not allow to address strategic variations among participants. More detailed kinematic analyses of the deceleration phase are necessary to address the repertoire of strategies, which is smaller in older than in young participants, with age-related differences in strategy performance and in sequential effects [59,60].

We used rather broad inclusion criteria and we did not perform systematic clinical assessments of the severity of patients’ sensorimotor impairment. As a consequence, we cannot easily link our results to clinical outcomes, which impedes any direct relation to possible clinical applications.

We did not separate participants with left vs. right lesion, and we did not take into account laterality before stroke. Consequently, the present results average the effects of consequences that differ among people with left and right stroke [15].

We acknowledge the diversity of the brain lesions among the 19 persons with stroke in the present study. Averaging over lesions hardly makes sense, and the detailed behavior of each patient differs from that of any other patient. For sure, the present approach will not serve for an individualized understanding of the functional consequences of the brain lesion. Yet, it is remarkable that, among this diversity, we were able to highlight regularities shared by all participants after a stroke, and that these regularities are also shared by age-matched controls and young adults representing the best possible human performance. Such regularities are likely the signatures of fundamental principles of sensorimotor control that are shared by people with stroke and control participants, but that are parameterized differently [61].

## Conclusion

Sensorimotor control after a stroke is characterized by higher uncertainty in the relation between the desired command and the effective motor output. Given a desired command, the uncertain motor output results in higher task-related variability, with consequences for trajectory formation, and especially for the partitioning between feed-forward and feed-back control.

Goal directed movements start with a feed-forward phase, eventually followed by feed-back corrections loops. Here, we found that the first sub-movement was designed to cover about 90% of the distance in young and age-matched healthy, while it was designed to cover about 45% of the distance after a stroke. We take this result as evidence that the same Bayesian decision rules apply for trajectory formation after a stroke and in healthy people: the weight of the feed-forward mode of control is inversely proportional to the uncertainty of its output. Because feed-forward is a reliable mode of control in healthy adults, the first sub-movement is designed to cover 90% of the distance to the target. Because feed-forward is an uncertain mode of control after a stroke, the first sub-movement is designed to cover 45% of the distance, and a series of sub-movements becomes mandatory to reach the target. The price to pay for the series of feed-back loops is a longer movement time, but it is only at this price that the accuracy constraint can be met.

## Supporting information

S1 DataSet Data used for the statistical analyses in the paper. (CSV)
